# Semi-continuous Propagation of Influenza A Virus and Its Defective Interfering Particles: Analyzing the Dynamic Competition To Select Candidates for Antiviral Therapy

**DOI:** 10.1128/JVI.01174-21

**Published:** 2021-11-23

**Authors:** Lars Pelz, Daniel Rüdiger, Tanya Dogra, Fadi G. Alnaji, Yvonne Genzel, Christopher B. Brooke, Sascha Y. Kupke, Udo Reichl

**Affiliations:** a Max Planck Institute for Dynamics of Complex Technical Systems, Bioprocess Engineering, Magdeburg, Germany; b University of Illinois at Urbana-Champaign, Department of Microbiology, Urbana, Illinois, USA; c Otto-von-Guericke-University Magdeburg, Bioprocess Engineering, Magdeburg, Germany; St. Jude Children’s Research Hospital

**Keywords:** influenza A virus, defective interfering particles, antiviral, next-generation sequencing, continuous virus production

## Abstract

Defective interfering particles (DIPs) of influenza A virus (IAV) are naturally occurring mutants that have an internal deletion in one of their eight viral RNA (vRNA) segments, rendering them propagation-incompetent. Upon coinfection with infectious standard virus (STV), DIPs interfere with STV replication through competitive inhibition. Thus, DIPs are proposed as potent antivirals for treatment of the influenza disease. To select corresponding candidates, we studied *de novo* generation of DIPs and propagation competition between different defective interfering (DI) vRNAs in an STV coinfection scenario in cell culture. A small-scale two-stage cultivation system that allows long-term semi-continuous propagation of IAV and its DIPs was used. Strong periodic oscillations in virus titers were observed due to the dynamic interaction of DIPs and STVs. Using next-generation sequencing, we detected a predominant formation and accumulation of DI vRNAs on the polymerase-encoding segments. Short DI vRNAs accumulated to higher fractions than longer ones, indicating a replication advantage, yet an optimum fragment length was observed. Some DI vRNAs showed breaking points in a specific part of their bundling signal (belonging to the packaging signal), suggesting its dispensability for DI vRNA propagation. Over a total cultivation time of 21 days, several individual DI vRNAs accumulated to high fractions, while others decreased. Using reverse genetics for IAV, purely clonal DIPs derived from highly replicating DI vRNAs were generated. We confirm that these DIPs exhibit a superior *in vitro* interfering efficacy compared to DIPs derived from lowly accumulated DI vRNAs and suggest promising candidates for efficacious antiviral treatment.

**IMPORTANCE** Defective interfering particles (DIPs) emerge naturally during viral infection and typically show an internal deletion in the viral genome. Thus, DIPs are propagation-incompetent. Previous research suggests DIPs as potent antiviral compounds for many different virus families due to their ability to interfere with virus replication by competitive inhibition. For instance, the administration of influenza A virus (IAV) DIPs resulted in a rescue of mice from an otherwise lethal IAV dose. Moreover, no apparent toxic effects were observed when only DIPs were administered to mice and ferrets. IAV DIPs show antiviral activity against many different IAV strains, including pandemic and highly pathogenic avian strains, and even against nonhomologous viruses, such as SARS-CoV-2, by stimulation of innate immunity. Here, we used a cultivation/infection system, which exerted selection pressure toward accumulation of highly competitive IAV DIPs. These DIPs showed a superior interfering efficacy *in vitro*, and we suggest them for effective antiviral therapy.

## INTRODUCTION

Yearly, on average, 400,000 people globally die from an infection with seasonal influenza A virus (IAV) (1). Moreover, the potential emergence of pandemic strains is a major threat to public health (2). The most effective prevention of the influenza disease is vaccination with tri- or quadrivalent formulations, which provide protection against different influenza virus strains (3, 4). However, influenza vaccines have to be reformulated annually as a result of antigenic drifts (5). This is associated with a potential decrease in vaccine efficacy due to false predictions and a vaccine mismatch to circulating strains (6). Furthermore, antiviral drugs targeting the viral neuraminidase (oseltamivir, zanamivir) (7) or the viral endonuclease (baloxavir) (8) may also be used. However, circulating strains have already shown resistance against available antivirals (9–11). Therefore, the development of effective prophylactic and therapeutic treatment options is urgently needed.

One promising approach for antiviral therapy is the application of defective interfering particles (DIPs) (12–16). These naturally occurring viral mutants feature an internal deletion in one of their eight viral RNA (vRNA) segments, which renders them defective in virus replication. In addition, a new species of IAV DIPs that showed point mutations on segment (Seg) 7 vRNA was discovered recently (17). DIPs can only replicate in a coinfection with infectious standard virus (STV), which complements the respective defect in the replication of the DIPs. These viral mutants are believed to interfere by preferential and faster replication of the defective interfering (DI) vRNA in comparison to the full-length (FL) vRNA, thereby drawing away cellular and viral resources required for STV growth (18–20). Furthermore, interference was shown at the packaging step, as DI vRNAs can selectively outcompete FL vRNA packaging (21, 22). Notably, in mouse and ferret models, the administration of DIPs resulted in a pronounced antiviral effect against IAV infection (13, 14, 23–26). Furthermore, IAV DIP treatment also resulted in protection against heterologous interferon (IFN)-sensitive respiratory virus infections (27, 28), including SARS-CoV-2 infection (29), by the ability of DIPs to enhance stimulation of innate immunity upon coinfection.

Recently, we established a two-stage bioreactor system for cell culture-based production of IAV (for vaccine manufacturing) (30) and production of a prototypic, well-characterized DIP (“DI244”) (23, 24, 27, 31). Here, uninfected cells (first bioreactor) were continuously fed to a second bioreactor that contained virus-infected cells. However, in such a continuous culture, the coinfection of STVs and DIPs typically results in periodic oscillations of virus titers due to their dynamic interactions. Moreover, *de novo* generation and accumulation of numerous DI vRNAs was observed (30, 31).

In the present study, a simplified, semi-continuous setup was used to thoroughly investigate the generation and growth competition between DIPs during 21 days of IAV infection. Assuming that DIPs showing exceptional propagation also show high interfering efficacies, we anticipated identification of potent candidates for antiviral therapy. For detection and quantification of the different deletion junction on the IAV vRNA level, we used a recently published next-generation sequencing (NGS) framework (32). We observed a small subset of highly accumulated DI vRNAs after 21 days postinfection (dpi), while other deletion junctions showed a pronounced decrease in their fractions in the same time frame. To generate corresponding purely clonal DIPs harboring the promising candidate DI vRNAs, we used reverse genetics for IAV. Indeed, these DIPs displayed a superior *in vitro* interfering efficacy compared to DIPs derived from lowly replicating DI vRNAs, indicating their potential for antiviral therapy.

## RESULTS

### Semi-continuous production of IAV results in periodic oscillations of virus titers and strong accumulation of DIPs.

**FIG 1:**
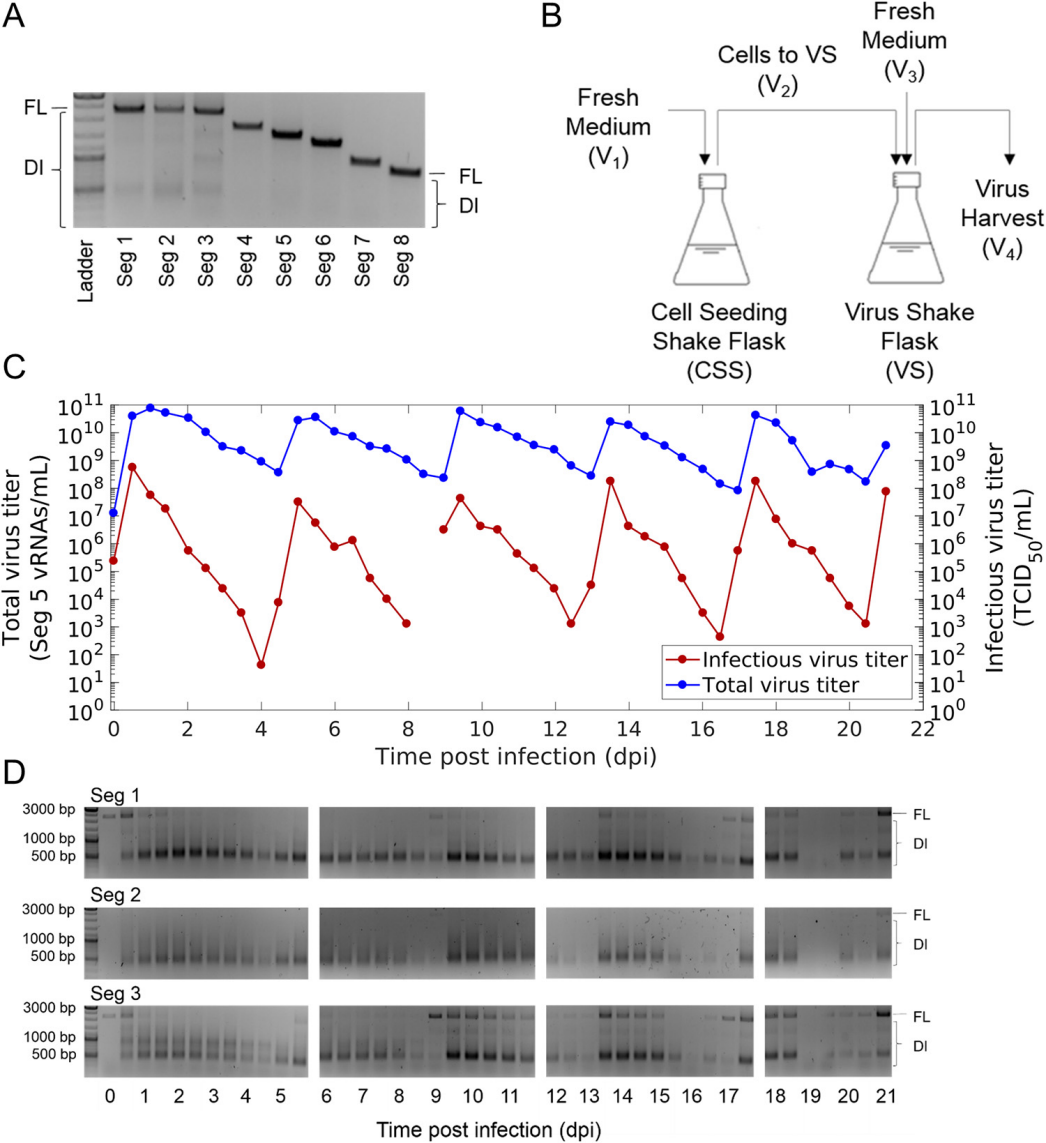
Semi-continuous propagation of influenza A virus and DIPs. (A) PR8 seed virus depleted in DI vRNAs was used for infection. Results of segment-specific RT-PCR for Seg 1 to 8 followed by agarose gel electrophoresis are shown. Signals corresponding to FL and DI vRNAs are indicated. The upper, middle, and lower thick bands of the DNA ladder indicate 3,000, 1,000, and 500 bp, respectively. (B) Experimental setup of the small-scale two-stage cultivation system in shake flasks (scheme adapted from Tapia et al. [68]). MDCK(sus) cells were grown in the CSS and VS. After an initial batch and semi-continuous phase (CSS and VS not coupled), the cells in the VS were infected with the seed virus (A) at an MOI of 0.1. The semi-continuous production mode was initiated 0.5 dpi, where cells were transferred from the CSS into the VS (V_2_) at regular time intervals, while fresh medium was added (V_1_ or V_3_) and virus harvest was taken for monitoring (V_4_). (C) Periodic oscillations of total and infectious virus titers during the production. The vRNA level of Seg 5 (indicating total virus particle concentration) was quantified by real-time RT-qPCR and infectious virus titer by TCID_50_ assay. (D) Accumulation of DI vRNAs over the semi-continuous production time of 21 days. Results of the segment-specific RT-PCR are shown for Seg 1, 2, and 3. Signals corresponding to FL and DI vRNAs are indicated. The illustration includes the results of one experiment.

In order to induce *de novo* generation and accumulation of DIPs, an IAV strain, A/PR/8/34, of the subtype H1N1 (PR8, provided by Robert Koch Institute, Berlin, Germany) was propagated in a semi-continuous small-scale two-stage cultivation system (Fig. 1B). For infection, we used a seed virus that was depleted in DI vRNAs as shown by segment-specific reverse transcription-PCR (RT-PCR) (Fig. 1A). Madin-Darby canine kidney (MDCK) cells growing in suspension culture (MDCK[sus]) were seeded into the cell seeding shake flask (CSS) and virus shake flasks (VS) at a viable cell concentration (VCC) of 0.6 × 10^6^ cells/ml and grown in batch mode to about 3.0 × 10^6^ cells/ml (–1.6 days postinfection [dpi]) (data not shown). Subsequently, for both shake flasks, a calculated volume of cell suspension was discarded, and fresh medium was added at regular time intervals (both shake flasks not yet connected in series). This resulted in a residence time (RT) of 38.3 h for both vessels. Note that preliminary studies showed a steady state in the VCC for this RT (data not shown). Once the steady state was reached, cells in the VS were infected with PR8 at a multiplicity of infection (MOI) of 0.1. At 0.5 dpi, both vessels were connected in series, and from there on, cells were transferred semi-continuously from the CSS to the VS (V_2_). In addition, fresh medium was added to both shake flasks (V_1_ or V_3_) and virus harvest was taken (V_4_). The RT chosen was 38.3 h and 22.0 h for CSS and VS, respectively, as this previously resulted in pronounced titer fluctuations and strong accumulation of DIPs (31). Over the production time of 21 dpi, the steady state in the CSS was kept with an average VCC of 2.6 × 10^6^ cells/ml (standard deviation [SD] of ±0.2 × 10^6^ cells/ml) (data not shown).

Strong periodic oscillations in the infectious virus titers (quantified by 50% tissue culture infective dose [TCID_50_] assay) and in the extracellular vRNA level of Seg 5 (quantified by real-time reverse transcription-quantitative PCR [RT-qPCR]) were observed in the VS (Fig. 1C). The extracellular vRNA level of Seg 5 was taken as a measure of the total virus concentration. DI vRNAs are mostly located on polymerase-encoding segments (20, 33–36), so the occurrence of DIPs should not affect the detection of Seg 5 vRNA. Shortly after infection at 0.5 dpi, a maximum infectious virus titer of 5.6 × 10^8^ TCID_50_/ml was reached. Here, high concentrations of STV (complying with a high MOI) increased the chance for coinfections with DIPs. Thus, a strong DIP propagation likely occurred early in cultivation, impeding STV propagation. Therefore, infectious virus titers decreased from 0.5 dpi onward. Eventually, the declining infectious virus titers led to fewer coinfections. Thus, DIP replication decreased, and the total virus particle concentration dropped as well. Additionally, DIPs were out-diluted because of the semi-continuous feeding strategy. Then, at a low infectious virus concentration (complying with a low MOI condition, ∼4.0 dpi), the chance of DIP coinfections was supposedly significantly reduced. Under these conditions, STVs could accumulate again as indicated by increasing virus titers toward 5 dpi. In the following, further periodic oscillations in virus titers occurred based on the DIP/STV interaction described above.

The dynamics in virus titers were well in agreement with results of the segment-specific RT-PCR (indicating FL and DI vRNAs) (Fig. 1D). A rapid accumulation of DI vRNAs occurred already at 0.5 dpi. Furthermore, the FL vRNA signal gradually dropped between 1 dpi and 2.5 dpi, suggesting the preferential production of DI vRNAs. Subsequently, DI vRNA replication decreased and DI vRNAs were washed out, as indicated by weaker band intensities of DI vRNAs (e.g., at 8.5 dpi). Next, in agreement with the increase of infectious viral titers (STVs), FL vRNA bands were visible again (e.g., at 9 dpi). Moreover, agarose gels indicated the presence of DI vRNA bands at the end of cultivation that may have been already present in the seed virus, suggesting that some DI vRNAs were preserved. In addition, weak DI vRNA bands as well as undefined, blurred bands emerged during the course of IAV replication, suggesting the formation and accumulation of *de novo*-generated DI vRNAs.

In summary, the semi-continuous production of IAV using a seed virus depleted in DI vRNAs led to the accumulation of DIPs. Thus, strong periodic oscillations in the total concentration of virions and infectious virus titers were observed due to the dynamic interaction of STVs and DIPs. Moreover, in the course of production, DIPs were exposed to high- and low-MOI conditions that likely resulted in alternating selection pressures, suitable for potential selection toward accumulation of highly interfering DIPs.

### Next-generation sequencing results indicate predominant *de novo* formation and accumulation of deletion junctions on polymerase-encoding segments.

Segment-specific RT-PCR does not enable the detection and quantification of individual deletion junctions. Therefore, to study the diversity of DI vRNAs generated during semi-continuous IAV propagation, samples were subjected to Illumina-based NGS and processed by a bioinformatics pipeline (32). In doing so, sequences of vRNAs from the produced progeny virions were obtained. Reads including a deletion junction (DI vRNA reads) do not align to the corresponding reference genome. These NGS reads were processed by the ViReMa algorithm to identify the position of individual deletion junctions (37).

**FIG 2:**
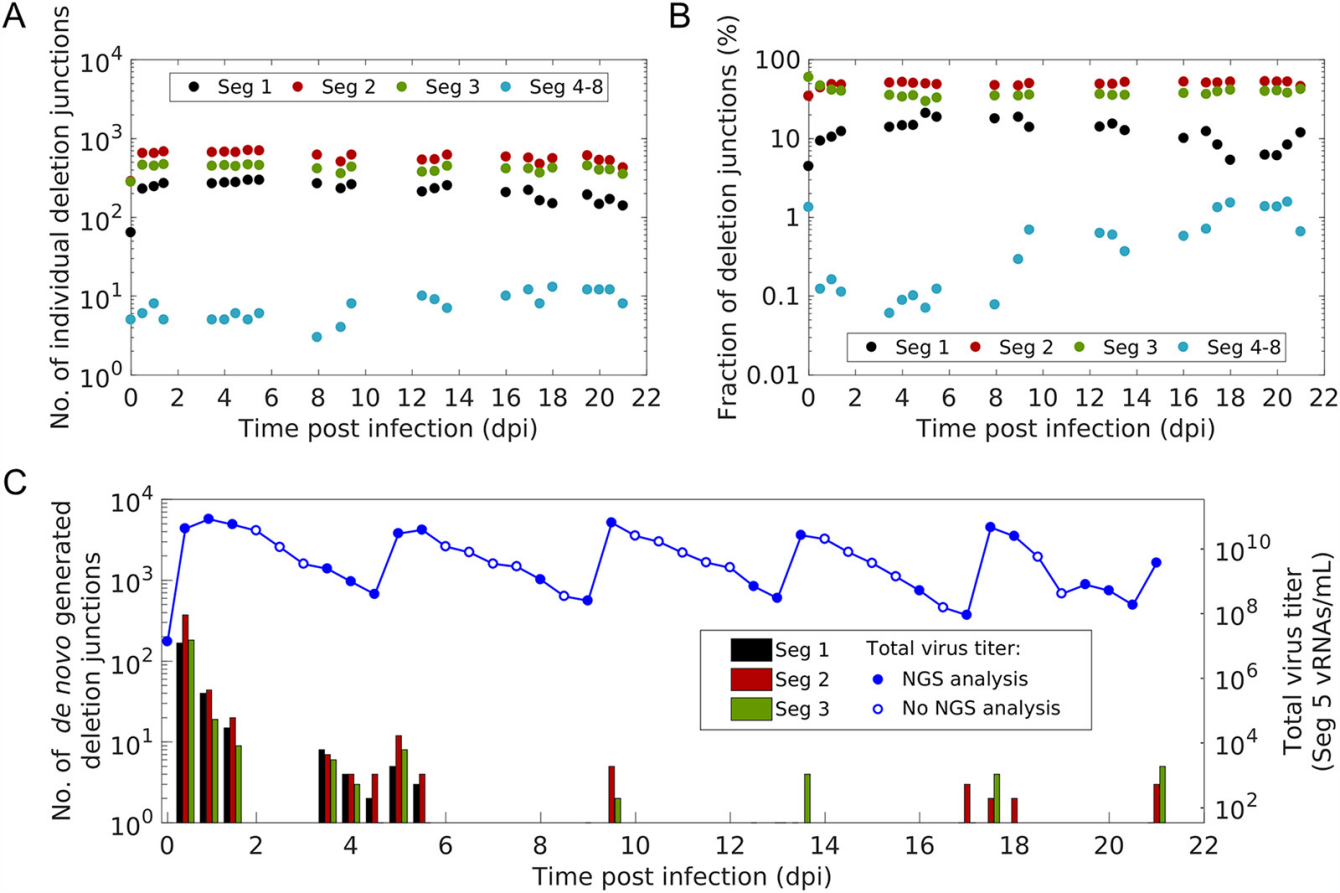
Diversity, distribution and *de novo* generation of deletion junctions during semi-continuous propagation of IAV. Deletion junctions were identified by Illumina-based NGS and subsequent analysis via the ViReMa algorithm (32). (A) Number of different deletion junctions located on the respective genome segment(s). (B) Fraction of all deletion junctions located on the respective genome segment(s). This fraction describes the ratio of the total number of detected deletion junctions for one segment to the total number of deletion junctions on all genome segments. (C) *De novo* formation of deletion junctions. The vRNA level of Seg 5 (indicating total virus particle concentration, as shown in Fig. 1C) was quantified by real-time RT-qPCR. Samples not analyzed by NGS are indicated by open circles. The illustration includes the results of one experiment.

The highest variation (i.e., number of different deletion junctions) was found on the polymerase-encoding segments 1 to 3, which encode the polymerase basic protein 2 (PB2) and 1 (PB1) and polymerase acidic protein (PA), respectively (Fig. 2A). Figure 2B shows the fraction of all deletion junctions located on a genome segment over time. Here, polymerase-encoding segments showed the highest fraction. In contrast, deletion junctions of non-polymerase-encoding segments showed a significantly lower fraction, which increased slightly toward the end of cultivation but always remained below 2%. As non-polymerase segment deletion junctions occurred only in negligible numbers, they were not considered any further in subsequent analyses.

Next, we investigated the *de novo* formation of DI vRNAs over the course of the cultivation. Figure 2C shows, at specific time intervals, the number of *de novo*-generated deletion junctions. *De novo* formation occurred mainly on the polymerase-encoding segments. Interestingly, most *de novo* formations occurred within the first 1.5 dpi. In addition, a considerable number of *de novo* DI vRNAs were detected between 3.5 and 5.5 dpi. However, *de novo* formation was significantly lower at later time points. Moreover, an increase in the number of new deletions was highly correlated with an increase in the total virus particle concentration (indicated by the vRNA level of Seg 5) (Fig. 2C). This is consistent with a fast STV replication and, thus, likely with a higher occurrence of the *de novo* formation of DI vRNAs due to the error-prone nature of the replication of the IAV RNA-dependent RNA polymerase.

In sum, our results show that DI vRNAs are predominantly *de novo* formed and accumulated on the polymerase-encoding segments during semi-continuous IAV infection.

### Short DI vRNAs tend to accumulate to higher fractions than longer ones; yet intermediate-length optima were observed as well.

**FIG 3:**
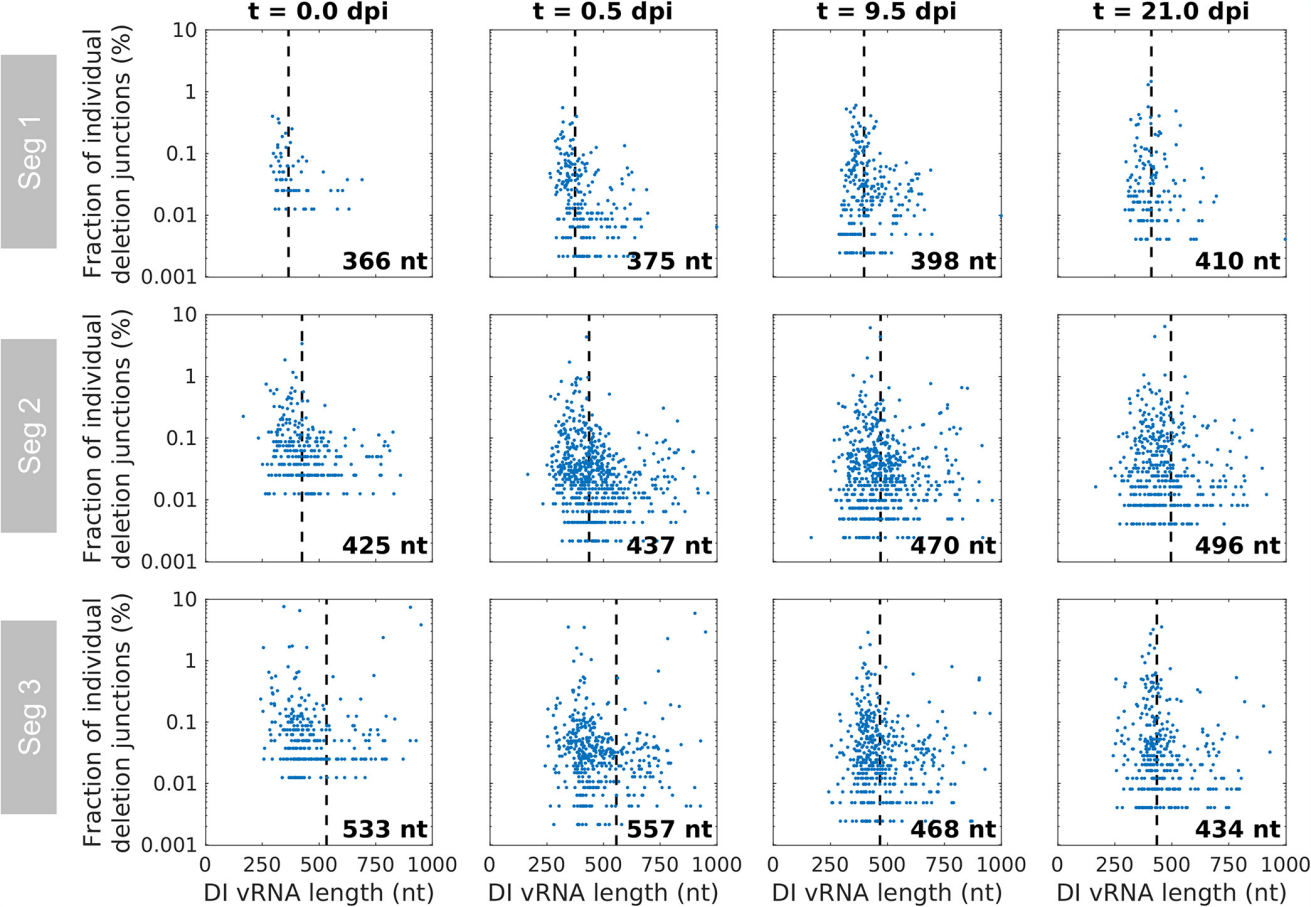
Dependency of the length of DI vRNAs on their accumulation during semi-continuous propagation of IAV. Deletion junctions were identified by Illumina-based NGS and subsequently analyzed via the ViReMa algorithm (32). Fractions of individual deletion junctions were calculated based on the ratio of the number of NGS reads of one individual deletion junction to the number of NGS reads of all deletion junctions located on all eight segments. The means of DI vRNA length (calculated by fitting a normal distribution function) are indicated by dashed vertical lines, and the corresponding lengths are shown. Representative time points are illustrated. The illustration includes the results of one experiment.

It was reported that DI vRNA accumulation surpasses that of FL vRNAs due to their shorter length resulting in a supposedly faster replication (12, 20). Therefore, we speculated that shorter DI vRNAs may also accumulate to higher abundances than longer DI vRNAs. Figure 3 shows the fraction of all individual deletion junctions and their corresponding DI vRNA length. Indeed, a bias toward accumulation of shorter DI vRNAs was observed, with short DI vRNAs showing overall higher fractions than longer ones during semi-continuous IAV production (Fig. 3). However, the highest fractions were not found for the shortest DI vRNAs. Rather, it appeared that the highest fractions were distributed around a length optimum. To visualize this optimum, we fitted a normal distribution function to the DI vRNA length and plotted the resulting mean as a dashed vertical line (Fig. 3). Over the whole cultivation, the mean DI vRNA length ranged between 366 and 414 nucleotides (nt), from 425 and 534 nt, and from 434 and 557 nt for Seg 1, 2, and 3, respectively.

Moreover, a few larger DI vRNAs (comprising a sequence length of up to 1,000 nt) accumulated to high fractions, suggesting that the sequence and the position of the deletion junction may be another factor to consider for replication of a DIP (Fig. 3). Note that Fig. 3 only shows DI vRNAs up to 1,000 nt in length, although we also detected very long DI vRNAs (>2,000 nt) (data not shown). These DI vRNAs with very short deletions may either not result in a defective vRNA, comprise two deletions, or represent technical artifacts. Due to their unknown origin and function and a lack of description in the literature, defective vRNAs larger than 85% of their respective FL length were excluded from analysis in this work.

Taken together, shorter DI vRNAs showed an overall stronger accumulation than longer DI vRNAs. However, highest fractions were distributed around an optimum length, indicating advantages for efficient DI vRNA replication and spreading.

### The incorporation signal but not the entire bundling signal appears to be required for propagation of DIPs.

**FIG 4:**
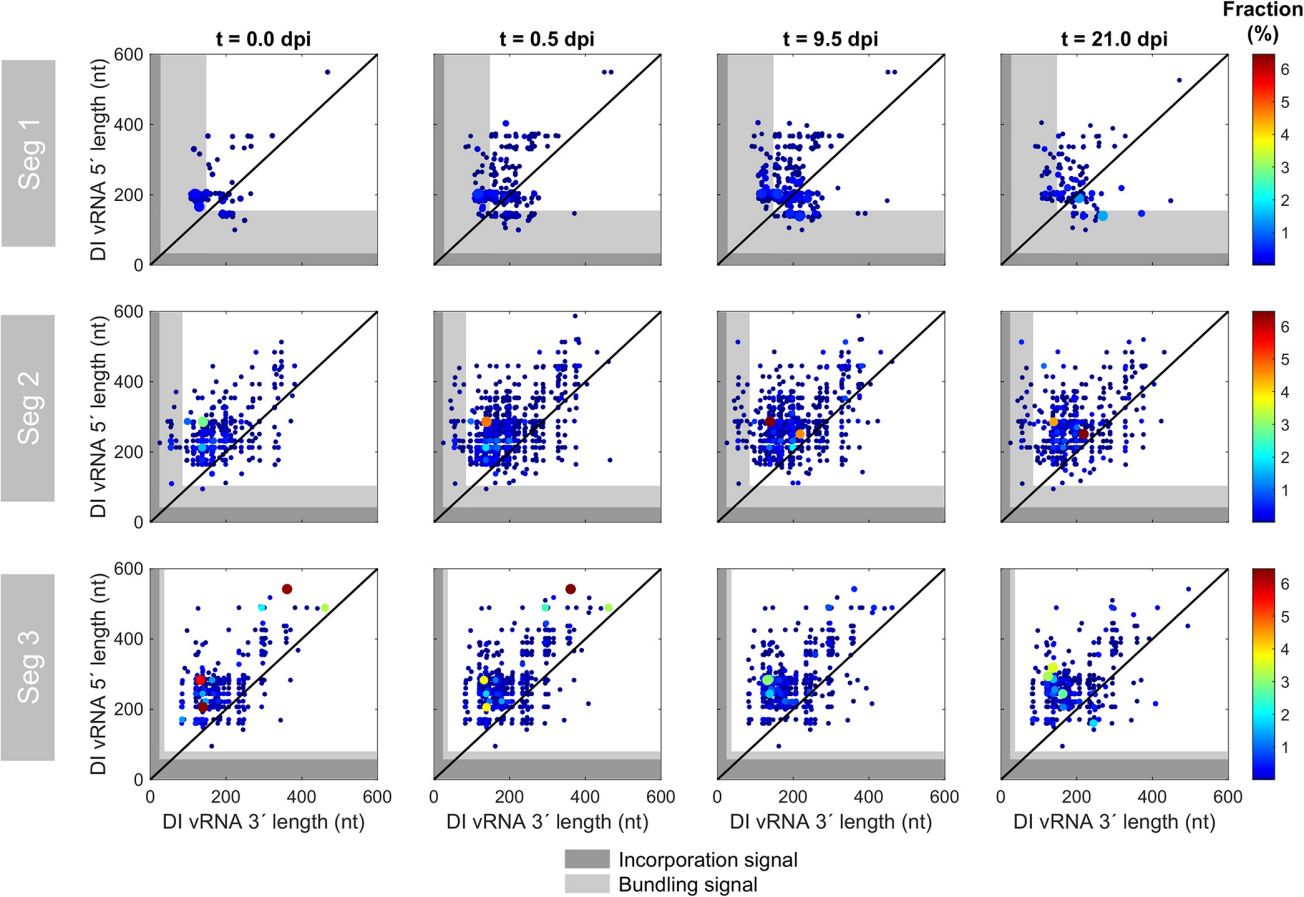
Deletion junction sites of DI vRNAs during semi-continuous propagation of IAV. Deletion junctions were identified by Illumina-based NGS and subsequently analyzed via the ViReMa algorithm (32). DI vRNA 3′ and 5′ lengths indicate the number of retained nucleotides prior to and after the deletion junction, respectively, at the corresponding vRNA ends. The packaging signal is indicated as gray areas and is divided into the incorporation signal (dark gray area) and bundling signal (light gray area). Representative time points are illustrated. The color code from red to blue shown on the right denotes the fraction of the individual deletion junction, which was calculated based on the ratio of the number of NGS reads of one individual deletion junction to the number of NGS reads of all deletion junctions located on all eight segments. Additionally, the circle radii increase with higher fractions. The diagonal black line indicates equal DI vRNA 3′ and 5′ lengths. The illustration includes the results of one experiment.

We next examined the position of the breaking points of DI vRNAs. Figure 4 illustrates the position of individual deletion junctions, as indicated by the number of retained nucleotides prior to (DI vRNA 3′ length) and after (DI vRNA 5′ length) the deletion junction site. In the course of the semi-continuous cultivation, breaking points were mostly located in proximity to both ends of vRNA (Fig. 4). This finding is in line with our observation of the predominant accumulation of short DI vRNAs (Fig. 3). We also observed highly abundant medium-sized DI vRNAs on Seg 3 in the seed virus (0.0 dpi), yet, the fraction of DI vRNAs carrying these deletions decreased, or even disappeared, toward the end of cultivation (Fig. 3). Again, this indicates that shorter DI vRNAs replicate faster and may outcompete longer ones. Additionally, the 3′ length of the DI vRNA largely did not correlate with the 5′ length, suggesting that deletion junctions are not preferably symmetrical.

**FIG 5:**
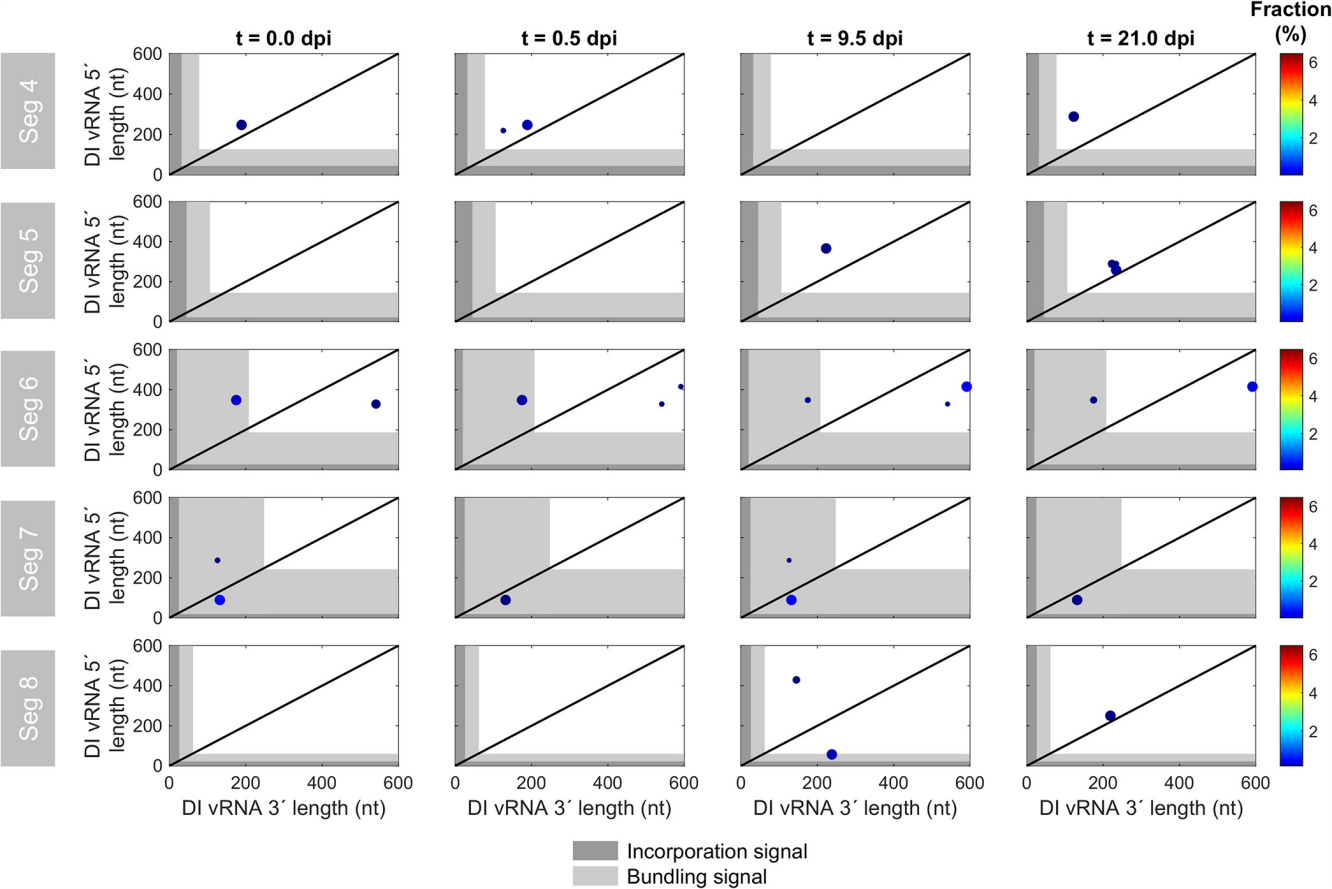
Deletion junction sites of DI vRNAs on non-polymerase-encoding segments during semi-continuous propagation of IAV. Deletion junctions were identified by Illumina-based NGS and subsequently analyzed via the ViReMa algorithm (32). DI vRNA 3′ and 5′ lengths indicate the number of retained nucleotides prior to and after the deletion junction, respectively, at the corresponding vRNA ends. The packaging signal is indicated as gray areas and is divided into the incorporation signal (dark gray area) and bundling signal (light gray area). Representative time points are illustrated. The color code from red to blue shown on the right denotes the fraction of the individual deletion junction, which was calculated based on the ratio of the number of NGS reads of one individual deletion junction to the number of NGS reads of all deletion junctions located on all eight segments. For graphs where no breaking points are shown, no DI vRNAs were detected at the selected time points. Additionally, the circle radii increase with higher fractions. The diagonal black line indicates equal DI vRNA 3′ and 5′ lengths. The illustration includes the results of one experiment.

While the lengths of the 3′ and 5′ ends ranged from below 100 nt to over 500 nt, specific minimum lengths were retained in the DI vRNAs (Fig. 4). We then asked whether the complete packaging signal (situated at the terminal ends of vRNA), which is important for organized packaging into progeny virions (38), was unaffected by deletions. A small percentage of breaking points was located in the packaging signal (on Seg 1 and 2), yet the majority of the deletion junction sites were located outside it, which is in line with the observation of an optimum in DI vRNA length (Fig. 3). For a more thorough investigation of deletion junctions in the packaging signal, we highlighted the positions of the incorporation signal (noncoding region [NCR], including the promoter region) and the bundling signal (terminal ends of the coding region) (39). The incorporation signal was reported to lead the packaging of the vRNA in which the signal is found. The second part of the packaging signal is the bundling signal, which confers the selective packaging of all the eight different segments together (39). We checked which part of the sequence at both ends was retained to infer a minimum sequence length for functional replication and packaging of the truncated vRNAs, assuming that only propagation-competent DI vRNAs can be detected. No deletion junctions in the incorporation signal for the polymerase-encoding segments or for Seg 4 to 8 were identified (Fig. 4 and 5, respectively). Therefore, we suggest that the preservation of the entire incorporation signal is crucial for the propagation of DIPs.

Interestingly, deletion junctions in the bundling signal (on Seg 1 and 2) could be detected, indicating that the entire bundling signal of these segments is most likely not required for propagation of DIPs. In particular, clusters of DI vRNA breaking points in the bundling signal were stable and present over the complete course of the semi-continuous cultivation. In contrast, Seg 3 did not show any breaking points in both signals. We found a minimum sequence length of 84 nt (3′ end) and 100 nt (5′ end), 25 nt and 95 nt, and 82 nt and 95 nt for Seg 1, 2, and 3, respectively. Figure 5 shows the position of deletion junction sites in Seg 4 to 8. Notably, although only very few individual deletion junctions were detected, breaking points were found in the bundling signal on Seg 6 (3′ end), Seg 7 (both ends), and Seg 8 (5′ end) as well.

In summary, our results indicate that the complete incorporation signal is crucial for propagation of DIPs, yet only a part of the bundling signal in Seg 1 and 2 seems to be required for DIP spreading.

### Dynamic competition in propagation between DI vRNAs leads to selection toward accumulation of highly interfering DIPs.

**FIG 6:**
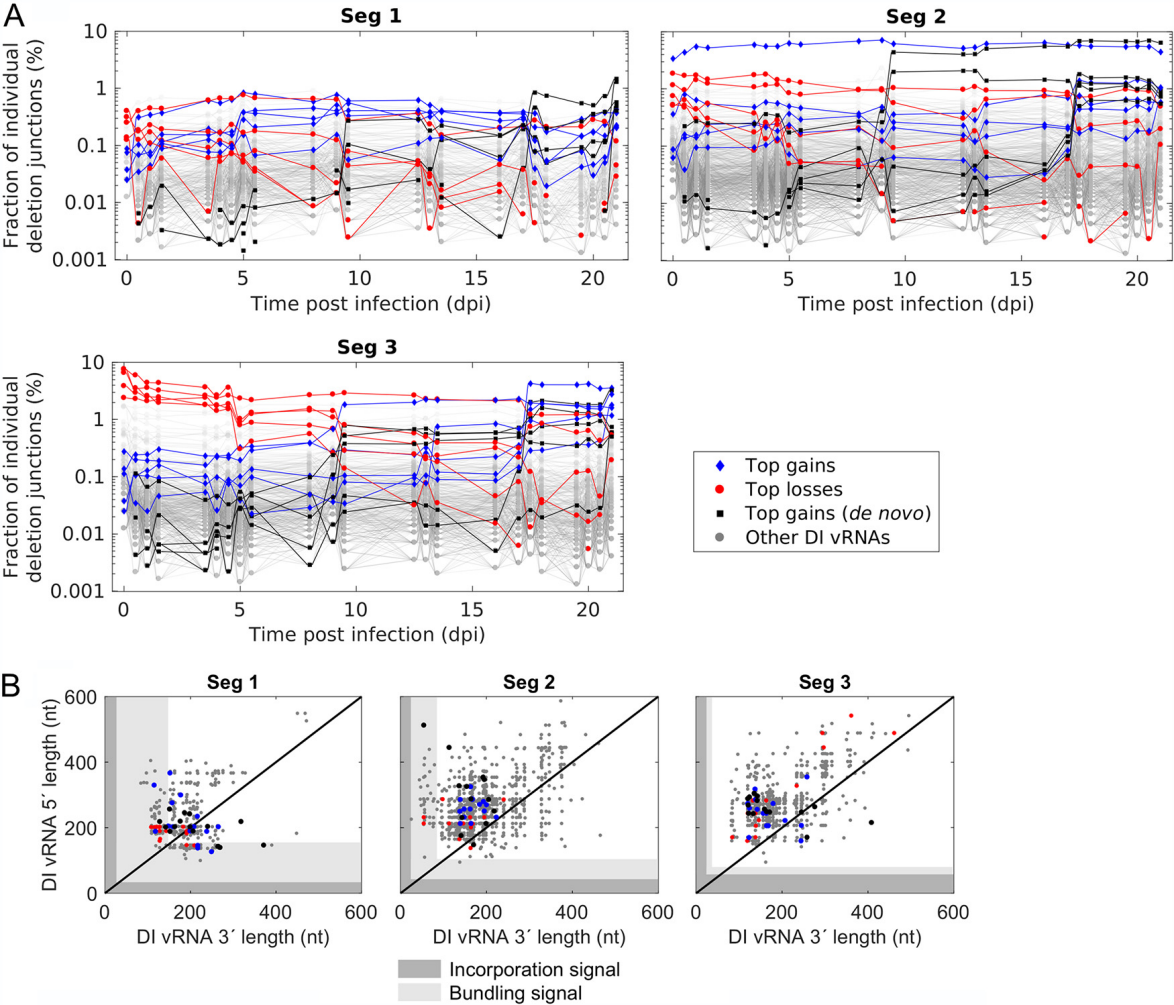
Propagation of DI vRNAs showing the highest gains or losses in their fractions during semi-continuous propagation of IAV. Deletion junctions were identified by Illumina-based NGS and subsequently analyzed via the ViReMa algorithm (32). Fractions of individual deletion junctions were calculated based on the ratio of the number of NGS reads of one individual deletion junction to the number of NGS reads of all deletion junctions located on all eight segments. (A) Fraction of individual DI vRNAs belonging to the group of top five gains, losses, and gains (*de novo*) of the fraction over cultivation time. Top gains (*de novo*) indicate newly formed deletion junctions with the highest fraction at the end of cultivation. (B) Deletion junction position of the top 15 gains, losses, and gains (*de novo*). DI vRNA 3′ and 5′ lengths indicate the number of retained nucleotides prior and after the deletion junction, respectively, at the corresponding vRNA ends. The packaging signal is indicated as gray areas and is divided into the incorporation signal (dark gray area) and bundling signal (light gray area). The illustration includes the results of one experiment.

In order to elucidate whether the various DI vRNAs show differences in their propagation, we next studied the composition of deletion junctions over cultivation time. More specifically, we determined the fraction of each individual deletion junction over time. Figure 6A shows these fractions and highlights the top five deletion junctions that showed the highest gain or largest loss in their fraction from the seed virus (0.0 dpi) to the end of cultivation (21 dpi). Likewise, the top five gains of *de novo*-formed DI vRNAs are indicated. Interestingly, differences between gains and losses were very pronounced, with a decreasing fraction of the top five losses, while the top five gains (including *de novo*) showed a strong accumulation. These trends were most prominent for Seg 3. Of note is also one deletion junction on Seg 2 that was present at a very high fraction in the seed virus and throughout the whole cultivation. Furthermore, pronounced shifts in the composition of deletion junctions were found for 9 to 9.5 and 17 to 17.5 dpi, at best visible for Seg 3. The occurrence of DI vRNAs that accumulate faster and achieve higher fractions than other DI vRNAs suggests that there was a dynamic competition in the propagation between individual DI vRNAs.

Moreover, we examined whether top gains (including *de novo*) and losses show differences in the deletion junction position (Fig. 6B). To obtain a better overview, we expanded the number of the top candidates in each category to 15. However, it appeared that no clear differences between the groups were present. For both gains and losses, few deletion junction sites were located in the bundling signal for Seg 1 and 2 (although most were found outside [Fig. 6B]), but none for Seg 3. Therefore, even for competitive DIPs (which require an efficient packaging process), we found a shorter packaging signal than that of the FL vRNA on Seg 1 (both ends) and on Seg 2 (3′ end). Please also note a few DI vRNAs (belonging to the top 15 losses) on Seg 3 showing a medium-sized DI vRNA length (∼900 nt) (Fig. 6B, upper right corner), which is in line with our observation that long DI vRNAs accumulate to low fractions (Fig. 3). However, we also found two top 15 gains (*de novo*) on Seg 2 with a very long DI vRNA (1,905 nt and 1,628 nt) (data not shown). This finding might suggest that not only the sequence length but also the breaking point position and probably further unknown regulatory effects are crucial for the efficient propagation of DI vRNAs.

In order to test the hypothesis, that fast-propagating DI vRNAs show a higher interfering efficacy than slow-propagating ones, we reconstituted the corresponding DIPs and tested them in an *in vitro* interference assay. More specifically, we rescued purely clonal DIPs (in the absence of STV) harboring either the top gain, top loss, or top gain (*de novo*) DI vRNA of Seg 1 (Table 1) using a modified reverse genetics system for IAV DIPs (25, 40). Next, we propagated these selected DIPs in genetically engineered MDCK-PB2(sus) cells, expressing PB2, to allow multiplication of these DIPs (harboring a deletion in Seg 1) without STV through complementation. Almost complete absence of contamination with other DI vRNAs was confirmed by results of segment-specific RT-PCR (data not shown).

**TABLE 1:** Generated Seg 1 candidate DIPs and the respective deletion junction positions in the 5′ to 3′ cDNA sequence

DIP	5′ Fragment size (bp)	3′ Fragment size (bp)	DIP fragment size (bp)
Loss	129	166	295
Gain	217	138	355
Gain (*de novo*)	269	140	409

**FIG 7:**
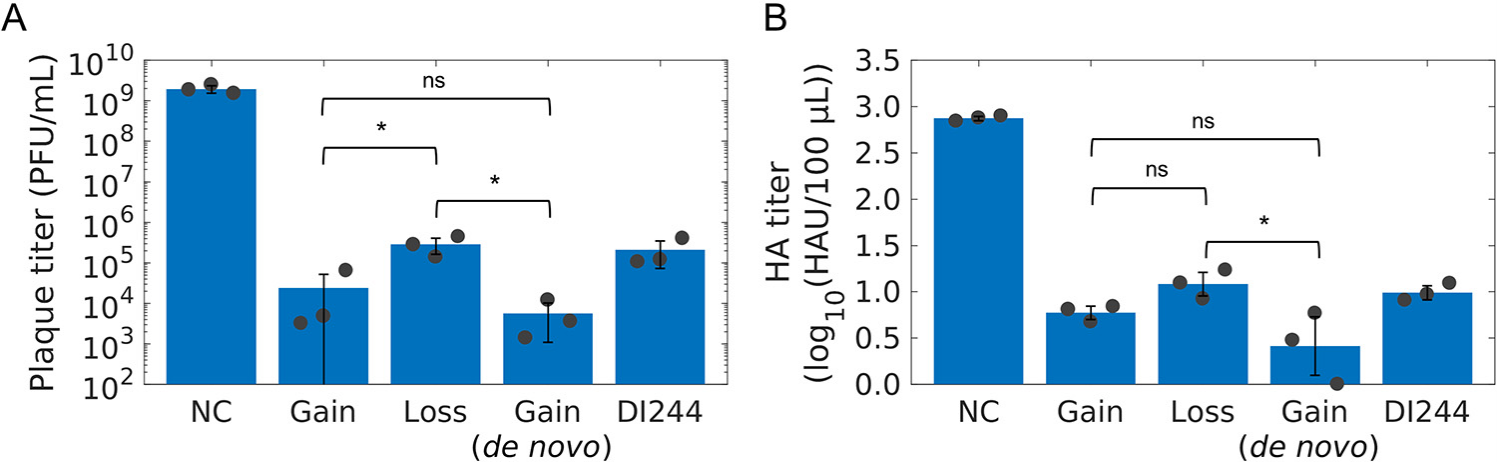
Interfering efficacy of DIPs derived from DI vRNAs showing the highest gain, loss, or gain (*de novo*) in their fraction during semi-continuous propagation of IAV. Purely clonal DIPs containing a deletion in Seg 1 (derived from DI vRNAs showing the top gains and top loss) were generated using a modified reverse genetics methodology for reconstitution of purely clonal IAV DIPs (40). Next, DIPs were multiplied in genetically engineered MDCK-PB2(sus) cells (expressing PB2) in a shake flask at a multiplicity of DIP (MODIP). (A and B) Interference assay. MDCK(adh) cells were infected with STV only at an MOI of 0.01 (NC) or coinfected with the corresponding DIP, resulting in a DIP/STV ratio of 3,224 (number of DIPs, 4.25 × 10^8^ virions; number of STVs, 1.32 × 10^5^ virions, derived from the HA titer). For comparison, DI244, a prototypic, well-characterized DIP (23–25, 27), was used. (A) Infectious virus release, quantified by plaque assay. (B) Total virus particle release, measured by HA assay. The illustration includes the results of three independent experiments (*n* = 3). Error bars indicate the SD. One-way analysis of variance (ANOVA) followed by Tukey’s multiple-comparison test (*, *P* < 0.05; ns, *P* > 0.05, not significant) was used to determine significance.

In the *in vitro* interference assay (Fig. 7), adherent MDCK cells (MDCK[adh]) cells were infected with STV at an MOI of 0.01 and coinfected with the different DIPs to evaluate the inhibition of STV replication compared to infection with STV alone (NC). Here, the DIP input for the interference assay was normalized through dilution based on the concentration of DIPs to ensure a direct comparison between the DIPs. In addition, we compared the interfering efficacy to a prototypic, well-characterized DIP named DI244 (23–25, 27). Indeed, the DIPs derived from the top gains (including *de novo*) showed the highest interfering efficacy. The top gain (*de novo*) DIP reduced the infectious virus release by more than 5 orders of magnitude, the top gain by 5 logs, while top loss and DI244 showed a reduction of only 4 orders of magnitudes (Fig. 7A). Reduction of the total virus particle release, indicated by the hemagglutination (HA) titer, showed a similar trend (Fig. 7B).

In summary, our results indicate that the semi-continuous propagation of IAV led to a dynamic competition in propagation between different DI vRNAs. We demonstrate that DI vRNAs showing the highest increase in the fraction over the cultivation period result in the formation of DIPs that show a superior interfering efficacy compared to DIPs containing slowly propagating DI vRNAs. These DIPs are, thus, promising candidates for antiviral therapy.

## DISCUSSION

IAV DIPs have been proposed as an effective antiviral agent for the influenza disease. In this study, we investigated the *de novo* generation and the competition in the growth between a diversity of DIPs during long-term semi-continuous IAV infection in order to identify strong candidates for antiviral therapy. In general, DIPs and STVs are in a competition for cellular and viral resources in a coinfection scenario (19, 41). Due to the replication advantage of DIPs, suppression of and interference with STV replication occurs (18–20). Moreover, it was shown that DIPs interfere with STV propagation at the packaging step, as preferential incorporation of DI vRNAs over FL vRNAs was observed (21, 22). We thus hypothesized that DI vRNAs showing the strongest accumulation during long-term coinfection possess the highest interference efficacy with STV replication. In our experiments, a small subset of individual DI vRNAs was observed that showed a pronounced accumulation, while the fractions of some other DI vRNAs strongly decreased (Fig. 6A). Next, DIPs harboring the most competitive DI vRNAs on Seg 1 were generated, and we showed that these DIPs exhibit a higher interfering efficacy than slowly propagating ones (Fig. 7). Strikingly, the interfering efficacy was also higher in comparison to DI244, a prototypic and well-characterized DIP (23–25), suggesting a huge potential of these candidates for antiviral treatment.

The antiviral mechanisms of IAV DIPs comprise (i) competitive inhibition during replication, i.e., “replication interference,” and (ii) inhibition of STV replication by the enhanced stimulation of innate immunity upon DIP coinfection (12, 42). Regarding the latter mechanism, the myxovirus resistance proteins (Mx) are thought to be a key player in the IFN-induced antiviral activity (43). However, in the present study, MDCK cells were used, where the canine Mx1 and Mx2 were shown to lack IFN-induced antiviral activity against IAV replication (44). In addition, trypsin (required for proteolytic activation of the viral hemagglutinin, enabling efficient multicycle replication) was used for virus propagation, yet it was shown that trypsin also strongly inhibits IFN signaling by proteolytic degradation of secreted IFNs (45). Therefore, the IFN response has presumably not significantly contributed to the oscillating virus replication in the present study. Our cultivation system has, rather, allowed for selection toward accumulation of DIPs showing a high “replication interference.” In the context of a potential antiviral treatment *in vivo*, note that retinoic acid-inducible gene I (RIG-I) preferentially associates with DI vRNAs over FL vRNAs (46), eventually resulting in an enhanced stimulation of the IFN response (47). We expect high DI vRNA accumulation in a coinfection with highly competitive DIPs (identified in this study), which may also result in a stronger stimulation of an antiviral innate immune response (besides their stronger “replication interference”) than coinfections with lowly accumulated DIPs. Corresponding experiments (*in vitro* and *in vivo*) are the subject of ongoing studies.

Our data show that the most competitive DI vRNAs are derived from the polymerase-encoding segments. Further, we found the highest variation, accumulation, and *de novo* formation of DI vRNAs on these segments (Fig. 2A, B, and C, respectively). This confirms previous studies which showed that DI vRNAs are predominantly found on Seg 1, 2, and 3 (20, 33–36). In agreement with this, a bias toward the emergence of DI vRNAs on the polymerase-encoding segments was observed during production of IAV over 17 days in a fully continuous two-stage bioreactor system (30). Mathematical modeling of the intracellular replication during STV and DIP coinfection also suggested that DI vRNAs located on the polymerase-encoding segments are more competitive than DI vRNAs on other segments (18). In particular, they even yielded high progeny numbers in less advantageous infection scenarios, i.e., when STV coinfection was delayed by several hours. Accordingly, in *in vivo* studies, DI244 containing a deletion on Seg 1, or DI vRNAs carrying a deletion in Seg 1, 2, or 3 showed a pronounced antiviral effect upon administration against IAV replication in mice and in ferrets (14, 23–25).

Our results show that short DI vRNAs tended to accumulate to higher fractions than longer DI vRNAs (Fig. 3). In general, it is believed that the shorter length of DI vRNAs (in comparison to FL vRNAs) leads to a replication advantage (18–20), which supports our findings. However, our observation of a length optimum also indicates that specific DI vRNA lengths are beneficial for DIP replication and spreading (Fig. 3). In agreement with this, the highly potent DI244 (395 nt) shows a similar DI vRNA length (Fig. 3) (23–25, 27). Other studies reported a similar mean DI vRNA length of 400 to 500 nt for Seg 1, 2, and 3 (12, 34). For DIPs originating from clinical isolates, similar mean DI vRNA lengths of 377 nt (Seg 1), 390 nt (Seg 2), and 490 nt (Seg 3) were found (36). Another investigation confirmed the finding of a replication advantage toward shorter vRNAs but additionally suggested that the sequence (untranscribed regions [UTRs] and coding region) may also have an influence on vRNA competition (41). This is consistent with previous work proposing that not only the length, but also the sequence (or the deletion junction position) may drive the replication advantage of DI vRNAs (20). This may explain our observation of few larger DI vRNAs up to 1,000 nt, which accumulated to high fractions (Fig. 3). In addition, two very long DI vRNAs (1,905 nt and 1,628 nt) were included in the top 15 gains (*de novo*) of DI vRNAs (data not shown). In further agreement, the *in vivo* interfering efficacy of three clonal DIPs containing DI vRNAs with a similar length (but diverse deletion junctions) differed significantly from each other (24). We found no clear patterns between the deletion junction positions of top gains (including *de novo*) and losses (Fig. 6B). These results may support the hypothesis that not only the DI vRNA length, but also the deletion junction site and further unknown regulatory effects are decisive factors for competitive DI vRNAs.

The packaging of progeny virions of IAV is a complex process. The leading packaging model postulates selective packaging of the eight different vRNA genome segments (48, 49). Decisive for correct and efficient packaging is a special vRNA sequence (packaging signal), which was discovered using reverse genetics approaches (38). As DIPs include a truncated segment, this packaging signal might equally be affected. However, it was suggested that this packaging signal in the shortened segment typically remains intact (12, 48). The packaging signal is divided into two parts, the incorporation signal (NCR, including promoter region) and the bundling signal (located at the terminal ends of the coding region). Our results show that the incorporation signal is crucial for DIP propagation, as it was unaffected by deletions (Fig. 4). However, several deletion junction sites were located in the bundling signal (Fig. 4, 6B, and 5). Therefore, we suggest that DIPs require only a part of the bundling signal for efficient replication and spreading. This finding does not agree with a previous study, which implied that the entire packaging signal is crucial for DI vRNA stability (50) and for high interference (51), yet only a few deletions were tested. The new insights obtained by our approach can be explained (i) by the significantly higher throughput of DI vRNA identification (in sum, on the order of 1,000) and (ii) by DIPs being challenged by alternating high- and low-MOI conditions for 21 days, which allowed extensive *de novo* generation and accumulation of new DIPs.

Previous work showed that DI vRNAs interfere with FL vRNAs during the packaging process, by selectively suppressing the packaging of either the parent segment (21, 22) or the FL vRNA of another genome segment (52). However, one recent study showed the opposite, in which DI vRNA were packaged less frequently than FL vRNA (previously posted on a preprint server [53]). Furthermore, differences in the packaging rates were found between individual DI vRNAs (21, 51). Thus, the highly abundant DI vRNAs found in the present study may have an advantage in the entire propagation process over others, including both replication and packaging. However, further in-depth studies are required to better characterize the interference of DI vRNAs at the virus assembly step.

Taken together, our findings show that DIPs containing DI vRNAs with a superior propagation rate also show a superior capacity to interfere with STV replication. These DIPs are very interesting candidates for antiviral treatment. The highly competitive DI vRNAs are predominantly located on the polymerase-encoding segments, display an optimal DI vRNA length, and conserve the incorporation signal but do not require the entire bundling signal. In addition, yet unidentified sequence motifs certainly also play an additional role during DI vRNA propagation. Due to the complex features of highly competitive DIPs, the best candidates for antiviral therapy are probably challenging to design *in silico*. Thus, evolution studies are a more convenient screening tool as shown for DIPs of other virus families (54–56).

## MATERIALS AND METHODS

### Cells and viruses.

MDCK(adh) cells (ECACC, No. 84121903) were adapted in previous works to grow in suspension culture (57) and then in chemically defined Xeno medium (58), in this work referred to as MDCK(sus) cells. Further, this cell line was engineered to stably express the PB2 for the production of purely clonal DIPs harboring a DI vRNA in Seg 1 (25, 40) and is denoted MDCK-PB2(sus). Cultivation of both cell lines was conducted in shake flasks at a working volume of 50 ml (125-ml baffled Erlenmeyer flask; Thermo Fisher Scientific, 4116-0125) using an orbital shaker (Multitron Pro, Infors HT; 50 mm shaking orbit) at 185 rpm and 37°C in a 5% CO_2_ environment. The medium was supplemented with 8 mM glutamine. For MDCK-PB2(sus) cells, puromycin (Thermo Fisher Scientific, no. A1113803) was added at a final concentration of 0.5 μg/ml. Quantification of VCC, viability, and diameter were performed using a cell counter (Vi-Cell XR; Beckman Coulter, no. 731050). MDCK(adh) cells were maintained in Glasgow minimum essential medium (GMEM; Thermo Fisher Scientific, no. 221000093) supplemented with 10% fetal bovine serum and 1% peptone at 37°C and 5% CO_2_. The corresponding adherent MDCK cell line that stably expressed PB2 [MDCK-PB2(adh)] (40) was maintained in the presence of 1.5 μg/ml puromycin. Adherent PB2-expressing HEK-293T (HEK-293T-PB2) cells (40) were maintained in Dulbecco’s modified Eagle medium (DMEM; Gibco, no. 41966029) supplemented with 10% fetal bovine serum, 1% penicillin streptomycin (10,000 units/ml penicillin and 10,000 μg/ml streptomycin; Gibco, no. 15140122), and 1 μg/ml puromycin at 37°C and 5% CO_2_.

For virus infection during semi-continuous cultivation, PR8 (provided by the Robert Koch Institute, Berlin, Germany) was used (58). The strain was adapted to MDCK(sus) cells, and depletion of DI vRNAs was carried out over five passages at a very low MOI of 10^−5^. For the interference assay in MDCK(adh) cells, the same PR8 strain, but adapted to adherent MDCK cells, was used. In addition, we generated candidate DIPs containing a deletion in Seg 1 using reverse genetics as described in “Generation of Purely Clonal DIPs Containing a Deletion in Seg 1,” below.

### Small-scale two-stage cultivation system for semi-continuous STV/DIP propagation.

For the semi-continuous propagation of PR8, a two-stage cultivation system was used, which consisted of two baffled shake flasks (250-ml baffled Erlenmeyer flask; Thermo Fisher Scientific, 4116-0250) connected in series (Fig. 1B). The CSS and the VS were operated at a working volume of 90.00 ml and 77.52 ml, respectively. MDCK(sus) cells in the exponential growth phase were seeded at a VCC of 0.6 × 10^6^ cells/ml and were cultivated in batch mode at 185 rpm and 37°C in a 5% CO_2_ environment for 2 days. When the VCC reached approximately 3.0 × 10^6^ cells/ml, at −1.6 dpi, a calculated volume of cell suspension was harvested every 12 h, while prewarmed fresh medium was added manually to obtain an RT (inverse of the dilution rate) of 38.3 h (both CSS and VS). Please note that both shake flasks were not yet connected in series. After steady state was achieved, the cells in the VS were infected with PR8 at an MOI of 0.1, and trypsin (Thermo Fisher Scientific, no. 27250-018) was added (final activity of 20 U/ml). At 12 h postinfection (hpi), semi-continuous production was started by transferring cells from the CSS to the VS (V_2_). Furthermore, virus was harvested (V_4_), and both shake flasks were filled with prewarmed fresh medium (V_1_ or V_3_) to obtain an RT of 38.3 h and 22.0 h for CSS and VS, respectively. It is important that the fresh medium, which was added to the VS, contained 60 U/ml trypsin to reach 20 U/ml in the VS. The respective transferred volumes are indicated (equations 1 to 4). The RT of 22.0 h for the VS was chosen, as previously published data showed a pronounced DIP/STV replication dynamic (31). In addition, samples were taken from the virus harvest at every volume transfer for analysis. Cell-free supernatants (300 × *g*, 4°C, 5 min) were stored at −80°C for further analysis.

(1) $$V_{1}=(t_{n}-t_{n-1})\cdot V_{\text{CSS}}\cdot D_{\text{CSS}}$$

(2) $$V_{2}=V_{1}=(t_{n}-t_{n-1})\cdot V_{\text{CSS}}\cdot D_{\text{CSS}}$$

(3) $$V_{3}=\left(t_{n}-t_{n-1}\right)\cdot (V_{\text{VS}}\cdot D_{\text{VS}}-V_{\text{CSS}}\cdot D_{\text{CSS}})$$

(4) $$V_{4}=\left(t_{n}-t_{n-1}\right)\cdot D_{\text{VS}}\cdot V_{\text{VS}}$$

where *t_n_* denotes the sample time point, and *t_n_*_–1_, the previous sample time point. *V*_CSS_ is the volume of the CSS, *V*_VS_ is the volume of VS, *D*_CSS_ is the dilution rate of CSS, and *D*_VS_ is the dilution rate of VS. *V*_CSS_, *D*_CSS_, and *D*_VS_ were predefined as mentioned above. V_3_ was set as 0.5 × V_2_ to ensure a sufficient volume of fresh medium in the VS. This assumption was applied to calculate the volume of *V*_Vs_.

### Virus quantification.

Quantification of the infectious virus titer was performed by TCID_50_ assay as described previously (59) with a measurement error of ± 0.3 log_10_ (60). The active DIP titer (required for calculation of a multiplicity of DIP [MODIP] for production of candidate DIPs in shake flasks; see “Rescue and Production of DIPs,” below) was quantified by plaque assay using MDCK-PB2(adh) cells (measurement error of ± 0.2 log_10_) (25). To determine the infectious virus titers in the interference assay (“Interference Assay,” below), MDCK(adh) cells were deployed in the same plaque assay (25). In addition, an HA assay was used to quantify the total number of virions in the supernatant with a measurement error of ±0.15 log_10_(HAU/100 μl) (61). The concentration of DIPs (*c*_DIP_) and concentration of STVs (*c*_STV_) were derived from the HA titer and determined according to equation 5, where *c*_RBC_ denotes the concentration of red blood cells (2.0 × 10^7^ cells/ml).

(5) $$c_{\text{DIP}}=10^{\frac{\log\mathrm{HA}}{100\text{μL}}}\cdot c_{\text{RBC}}$$

### PCR measurements.

Genomic vRNA in progeny virions was examined using PCR. In brief, isolation of vRNA from 150 μl cell-free supernatants was carried out with the NucleoSpin RNA virus kit (Macherey-Nagel, 740956) as described in the manufacturer’s instructions. In order to analyze the presence of FL vRNA and DI vRNA (truncated form), a segment-specific RT-PCR was performed (see “Segment-Specific RT-PCR,” below). Real-time RT-qPCR was applied for absolute quantification of Seg 5 vRNA from the progeny virions (see “Real-Time RT-qPCR,” below).

**Segment-specific RT-PCR.** A recently described method was utilized for segment-specific RT-PCR (17, 30). In brief, isolated vRNA was reverse transcribed into cDNA using universal primers that bind at the conserved terminal regions of all eight IAV genome segments (Table 2). Subsequently, segment-specific primers were used for amplification of the respective genome segment sequence by PCR (Table 2). Finally, PCR products were analyzed by agarose gel electrophoresis.

**TABLE 2:** Primers used for segment-specific RT-PCR

Reaction	Target	Primer name	Sequence (5′→3′)
RT	All segments	Uni 12	AGCAAAAGCAGG
PCR	Segment 1	S1 Uni for	AGCGAAAGCAGGTCAATTAT
		S1 Uni rev	AGTAGAAACAAGGTCGTTTTTAAAC
	Segment 2	S2 Uni for	AGCGAAAGCAGGCAAACCAT
		S2 Uni rev	AGTAGGAACAAGGCATTTTTTCATG
	Segment 3	S3 Uni for	AGCGAAAGCAGGTACTGATCC
		S3 Uni rev	AGTAGAAACAAGGTACTTTTTTGG
	Segment 4	S4 Uni for	AGCAAAAGCAGGGGAA
		S4 Uni rev	AGTAGAAACAAGGGTGTTTT
	Segment 5	S5 Uni for	AGCAAAAGCAGGGTAGATAATC
		S5 Uni rev	AGTAGAAACAAGGGTATTTTTC
	Segment 6	S6 Uni for	AGCGAAAGCAGGGGTTTAAAATG
		S6 Uni rev	AGTAGAAACAAGGAGTTTTTTGAAC
	Segment 7	S7 Uni for	AGCGAAAGCAGGTAGATATTG
		S7 Uni rev	AGTAGAAACAAGGTAGTTTTTTAC
	Segment 8	S8 Uni for	AGAAAAAGCAGGGTGACAAA
		S8 Uni rev	AGTAGAAACAAGGGTGTTTT

**Real-time RT-qPCR.** A recently reported method for the specific detection and quantification of influenza viral RNA segments using real-time RT-qPCR was employed (17, 62, 63). Briefly, RNA reference standards were *in vitro* synthesized for absolute quantification (primers required for generation are listed in Table 3). Isolated vRNA of the samples was used for reverse transcription, along with a dilution series of the reference standards (primers listed in Table 4), followed by real-time qPCR (primer sequence in Table 5). Calculation for absolute quantification of vRNA of Seg 5 was conducted as previously described (17, 63).

**TABLE 3:** Primers used for reference standard generation

Target	Primer name	Sequence (5′→3′)
Seg 5	S5 uni for	AGCAAAAGCAGGGTAGATAATC
	S5 uni T7 rev	TAATACGACTCACTATAGGGAGTAGAAACAAGGGTATTTTTC

**TABLE 4:** Primers used for RT

Target	Primer name	Sequence (5′→3′)
Seg 5	S5 tagRT for	ATTTAGGTGACACTATAGAAGCGAGTGATTATGAGGGACGGTTGAT

**TABLE 5:** Primers used for real-time qPCR

Target	Primer name	Sequence (5′→3′)
Introduced tag sequence	vRNA tagRealtime for	ATTTAGGTGACACTATAGAAGCG
Seg 5	Seg 5 Realtime rev	CGCACTGGGATGTTCTTC

**NGS and data processing.** Sample preparation, NGS library preparation, and sequencing analysis of deletion junctions was performed according to a recently published study (32).

### Analysis of deletion junctions.

Deletion junctions refer to the DI vRNAs in the viral population, while deletion junction sites refer to the start and end position of the breaking points in the viral genome. Deletion junctions that did not accumulate to levels above 14 NGS reads in at least one sampling time point were removed from the data set for higher accuracy (32). Furthermore, defective vRNAs that showed more than 85% of the length of FL vRNA were excluded from analysis in this work. DI vRNA 3′ and 5′ length indicated the number of retained nucleotides prior and after the deletion junction at the respective vRNA end. Of note is that the DI vRNA sequence was reported in negative-sense and 3′ to 5′ orientation. The calculation of the DI vRNA length comprised the following sequence lengths: Seg 1 (2,341 nucleotides [nt]), Seg 2 (2,341 nt), Seg 3 (2,233 nt), Seg 4 (1,775 nt), Seg 5 (1,565 nt), Seg 6 (1,413 nt), Seg 7 (1,027 nt), and Seg 8 (890 nt). The number of nucleotides of the incorporation signal (NCR) and the bundling signal (terminal ends of coding region), which together form the packaging signal, were taken from a recent review (64).

### Generation of purely clonal DIPs containing a deletion in Seg 1.

To generate purely clonal Seg 1-derived DIPs (top gain, loss, gain [*de novo*]) in the absence of STV, we used a previously established plasmid-based reverse-genetics system (40). More specifically, to complement the missing PB2 to allow DIP production without STV, we used a coculture of HEK-293T-PB2 cells and MDCK-PB2(adh) cells for reconstitution.

**Generation of plasmids.** Plasmids harboring specific deletions were generated as described previously (40). In brief, pHW191 encoding the PR8-derived PB2 gene (65) was used as a template for PCR amplification (Phusion Hot Start II DNA polymerase; Thermo Fisher, no. F549L). Here, the desired 5′ fragment (containing overhangs complementary to the 3′ fragment) of a specified deletion junction (Table 1) was amplified, using a 5′-specific forward and reverse primer set (Table 6). Similarly, a set of 3′-specific primers were used to amplify the desired 3′ fragment (containing overhangs complementary to the 5′ fragment) of a specified deletion junction from the pHW191 template DNA. Next, the 5′ fragments hybridized with the overlapping 3′ fragments, resulting in PCR products with the individual deletion junctions (splice-overlapped products) after subsequent amplification cycles at an annealing temperature of 62°C. Lastly, the internally spliced PB2 sequence was inserted in pHW2000-GGAarI using Golden Gate cloning (66, 67). All plasmids were sequenced to confirm the generated deletion junctions.

**TABLE 6:** Splice overlap extension PCR primers

Target	Primer name	Sequence (5′→3′)	Annealing temp for overlap PCR (°C)
Gain 5′ fragment	For	CGGTCACCTGCCAGTGGGAGCGAAAGCA	66
	Rev	ACGTCTCCTTGCCCAATTATCCTCTTGTCTGCTGTA	
Gain 3′ fragment	For	TACAGCAGACAAGAGGATAATTGGGCAAGGAGACGT	
	Rev	CCCACCTGCGCGCTATTAGTAGAAACAAGGTCGTTTTTAAACTATT	
Loss 5′ fragment	For	CCCACCTGCCAGTGGGAGCGAAAGCAG	67
	Rev	GCCTTCTCTCCTTTCGCGTACTTCTTGATTATGGCCA	
Loss 3′ fragment	For	TGGCCATAATCAAGAAGTACGCGAAAGGAGAGAAGGC	
	Rev	CCCACCTGCGCGCTATTAGTAGAAACAAGGTCGTTTTTAAACTA	
Gain (*de novo*) 5′ fragment	For	CAGTCACCTGCCGATGGGAGCGAAAGCAGGT	67
	Rev	CCACGTCTCCTTGCCCAATTATTTTACTCCATAAAGTTTGTCCTTGC	
Gain (*de novo*) 3′ fragment	For	GCAAGGACAAACTTTATGGAGTAAAATAATTGGGCAAGGAGACGTGG	
	Rev	CCCACCTGCTTTTTATTAGTAGAAACAAGGTCGTTTTTAAACTATTC	

**Rescue and production of DIPs.** For rescue of purely clonal DIPs containing a deletion in Seg 1 (40), we cotransfected a coculture of adherent HEK-293T-PB2 cells (0.2 × 10^6^ cells/well) and MDCK-PB2(adh) cells (0.2 × 10^6^ cells/well) with corresponding plasmids harboring a deletion in the PB2 sequence (50 ng) and 1 μg of each pHW192-pHW198 plasmid (encoding the remaining gene segments of PR8 IAV) using the calcium phosphate method in a 6-well format. DIP-containing supernatants were harvested at 4, 6, 8, 10, and 12 days posttransfection and stored at −80°C for further use. Larger stocks (seed viruses) of purely clonal Seg 1 DIPs were generated in MDCK-PB2(sus) cells in shake flasks.

The production of Seg 1 DIPs in MDCK-PB2(sus) cells was conducted according to a recently published paper (25). In brief, MDCK-PB2(sus) cells cultivated in shake flasks were centrifuged (300 × *g*, 5 min, room temperature) and used to inoculate a new shake flask at 2.0 × 10^6^ cells/ml with fresh medium and trypsin (final activity of 20 U/ml). Subsequently, cells were infected at an MODIP of E-2. Cell-free supernatants (3,000 × *g*, 4°C, 10 min) were stored at −80°C for further analysis.

### Interference assay.

To measure the efficacy of DIPs to suppress STV replication, we performed an *in vitro* coinfection assay in MDCK(adh) cells following a previously published description (26). To summarize, MDCK(adh) cells, cultivated in 6-well plates, were washed twice with phosphate-buffered saline (PBS). Next, cells were either infected with STV only (MOI of 0.01, based on the TCID_50_ titer) or coinfected with STV and 125 μl of the produced DIP material (diluted for normalization). Wells were filled up to 250 μl with infection medium (GMEM; 1% peptone, 5 U/ml trypsin), and incubation was conducted for 1 h at 37°C and 5% CO_2_. Subsequently, the inoculum was aspirated, the cells washed with PBS, and 2 ml of infection medium was added. Cells were incubated for 24 h at 37°C and 5% CO_2_. The supernatant was harvested and stored at −80°C until further analysis by plaque assay and HA assay.

### Data availability.

The reference sequence of the PR8 genome used for alignment can be found under the following NCBI accession numbers: AF389115.1 (PB2), AF389116.1 (PB2), AF389117.1 (PA), AF389118.1 (HA), AF389119.1 (NP), AF389120.1 (NA), AF389121.1 (M), AF389122.1 (NS). The complete NGS data set is available under the BioProject accession number PRJNA743179.
